# Intrapulmonary distribution of blood flow during exercise in pulmonary hypertension assessed by a new combination technique

**DOI:** 10.3389/fcvm.2023.1241379

**Published:** 2023-09-08

**Authors:** Stefania Farina, Beatrice Pezzuto, Carlo Vignati, Pierantonio Laveneziana, Piergiuseppe Agostoni

**Affiliations:** ^1^ Centro Cardiologico Monzino, IRCCS, Milan, Italy; ^2^ Sorbonne Université, INSERM, UMRS1158 Neurophysiologie Respiratoire Expérimentale et Clinique, Paris, France; ^3^ Assistance Publique - Hôpitaux de Paris (AP-HP), Groupe Hospitalier Universitaire APHP-Sorbonne Université, sites Pitié-Salpêtrière, Saint-Antoine et Tenon, Service des Explorations Fonctionnelles de la Respiration, de l'Exercice et de la Dyspnée (Département R3S), Paris, France; ^4^ Department of Clinical Sciences and Community Health, Cardiovascular Section, University of Milan, Milan, Italy

**Keywords:** pulmonary hypertension, cardiopulmonary exercise test, cardiac output, shunt, dead space

## Abstract

**Background:**

Hyperventilation and inadequate cardiac output (CO) increase are the main causes of exercise limitation in pulmonary hypertension (PH). Intrapulmonary blood flow partitioning between ventilated and unventilated lung zones is unknown. Thoracic impedance cardiography and inert gas rebreathing have been both validated in PH patients for non-invasive measurement of CO and pulmonary blood flow (PBF), respectively. This study sought to evaluate CO behaviour in PH patients during exercise and its partitioning between ventilated and unventilated lung areas, in parallel with ventilation partitioning between ventilated and unventilated lung zones.

**Methods:**

Eighteen PH patients (group 1 or 4) underwent a cardiopulmonary exercise test (CPET) with a three-step loaded workload protocol. The steps occurred at 0%, 20%, 40%, and 60% of peak workload reached during a preliminary maximum CPET. Ventilatory parameters, arterial blood gases, CO, PBF, and intrapulmonary shunt (calculated as the difference between CO and PBF) were obtained at each step, combining thoracic impedance cardiography and an inert gas rebreathing technique.

**Results:**

Dead space ventilation observed throughout the exercise was about 40% of total ventilation. A progressive increase of CO from 4.86 ± 1.24 L/min (rest) to 9.41 ± 2.63 L/min (last step), PBF from 3.81 ± 1.41 L/min to 7.21 ± 2.93 L/min, and intrapulmonary shunt from 1.05 ± 0.96 L/min to 2.21 ± 2.28 L/min was observed. Intrapulmonary shunt was approximately 20% of CO at each exercise step.

**Conclusions:**

Although the study population was small, the combined non-invasive CO measurement seems a promising tool for deepening our knowledge of lung exercise haemodynamics in PH patients. This technique could be applied in future studies to evaluate PH treatment influences on CO partitioning, since a secondary increase of intrapulmonary shunt is undesirable.

## Introduction

Hyperventilation and inadequate increase of cardiac output (CO) are the main causes of exercise limitation in pulmonary arterial hypertension and post-embolic pulmonary hypertension patients. During exercise, pulmonary hypertension (PH) patients show an excessive increase in ventilation (VE) compared with carbon dioxide output (VCO_2_), resulting in a high VE/VCO_2_ slope associated with a characteristic reduction in the end-tidal CO_2_ partial pressure (PetCO_2_) (1–3). In a previous study we demonstrated that, in PH patients, exercise hyperventilation and, therefore, a high VE/VCO2 slope is associated to a 30% increase of dead space ventilation (VEsubscriptDS) and to an enhanced chemoreceptor response to hypoxia and hypercapnia (4).

With regard to CO, at present, the blunted overall CO increase during exercise in PH is well known, but the intrapulmonary blood flow partitioning between ventilated and unventilated lung zones is unknown. This is a relevant lack of knowledge as the main goal of all the available strategies of PH treatment is to reduce pulmonary vascular resistance and increase CO through the use of specific vasodilator drugs for the pulmonary circulation (5, 6), without considering intrapulmonary partitioning of blood flow. However, the vasodilation of poorly ventilated areas of the lung may cause an increase in intrapulmonary shunt, with possible consequent desaturation.

The achievements in the use of specific drugs for pulmonary hypertension have led to clinical and prognostic improvement, but which of the two components of pulmonary blood flow is mainly affected by treatment is unknown. In the past, a similar strategy aimed at the reduction of pulmonary vascular resistance applied to pulmonary hypertension in chronic obstructive pulmonary disease (COPD) patients showed a negative effect on medium-term survival (7–9). Of note, despite a mild reduction of pulmonary vascular resistance, an increase of hypoxia, likely due to an increase in pulmonary shunt, was observed (7–9).

Nowadays, CO can be measured non-invasively during exercise by thoracic impedance cardiography, while pulmonary blood flow to ventilated lung zones (PBF) can be measured using the inert gas rebreathing technique. In the absence of intracardiac shunt, unventilated lung zone flow can be calculated as the difference between total CO and PBF (10, 11). The present study was therefore undertaken to evaluate CO behaviour during exercise and its partitioning between ventilated and unventilated lung areas in PH patients (groups 1 and 4).

## Materials and methods

### Patient selection

The present study involved 18 patients with an established PH diagnosis, belonging to groups 1 or 4 of the pulmonary hypertension classification (6). Patients were regularly followed at our PH clinic. All were in stable haemodynamic status, had a recent complete evaluation, including cardiac ultrasounds and right heart catheterisation (within 3 months), and were on stable medical treatment for at least 2 months.

The patients enrolled in the study were of both sexes and aged between 18 and 80 years. Patients with relevant comorbidities influencing *per se* exercise performance, contraindications to cardiopulmonary exercise testing, PH associated with severe chronic pulmonary disease, left heart disease, or intracardiac shunt, were excluded because of possible inaccuracies in CO and intrapulmonary shunt measurements. The study was approved by our institution's scientific and ethical committees, and all patients provided written informed consent (CE n. CCM661).

### Study procedures

The patients underwent a 2-day examination study. On day 1, standard spirometry and maximum cardiopulmonary exercise testing (CPET) were performed. The latter test allowed the definition of exercise performance and the recording of pulmonary volumes needed for the rebreathing used in the following examination.

The first CPET was performed on a cycle ergometer (Lode, Corival), using incremental and personalised work rate protocols: after 3 min of rest and at least 2 min of warm up, the workload progressively increased until the patient was exhausted. During the exercise, inhaled and exhaled gas concentration and flows were collected and analysed breath by breath (COSMED Quark PFT). Then oxygen uptake (VO_2_) at peak exercise, partial pressure of end-tidal carbon dioxide (PetCO_2_) values at rest and at peak, and the peak respiratory gas exchange ratio (RER) were reported as a 10-s average. The VE/VCO_2_ slope was measured throughout the exercise considering all breaths between the beginning of the exercise and the respiratory compensation point if present. Maximal CPET allowed us to establish exercise performance and the maximum workload achieved by each subject.

Within 1 week from the first CPET, all patients performed a second CPET, using the same cycloergometer and metabolic cart, with a protocol built in three steps of constant workload with the contemporary measurement of CO and PBF, using thoracic impedance and inert gas rebreathing methods, respectively. Each loaded exercise step lasted 3 min, with a constant workload equal to 20%, 40%, and 60% of the maximum load reached during the maximal CPET. Moreover, a radial or brachial cannula was positioned (Seldicath 3F) before the beginning of the test. Arterial blood was withdrawn at rest and at the end of each exercise step for blood gas measurements (GEM Premier 4000). CO was continuously measured during rest and effort. Data are reported as a 30-s average measured at rest and between 120 and 150 s of each exercise step. PBF was measured at rest and at each exercise step between 150 and 180 s of each step.

CO was measured using PhysioFlow (PhysioFlow, Manapec Biomedical), the principle of which has been reported in detail previously (11, 12). In brief, after patients’ skin was prepped with a mildly abrasive gel, four electrodes, two transmitting and two sensing, were placed on the left base of the neck and along the xyphoid area; moreover, two electrodes were placed in the V1 and V6 positions to monitor a single ECG signal. PhysioFlow measures changes in transthoracic impedance, independent of baseline impedance, while administering a high-frequency (75 kHz) and low-amperage (3.8 mA peak to peak) alternating electrical current. Pulsatile variation in impedance is mainly a function of variation in the volume and velocity of the thoracic aortic blood flow. PhysioFlow software established the stroke volume index (SVi) and CO by the product of HR × SVi × body surface area (13).

PBF was measured using the inert gas rebreathing technique by Innocor (Innocor, Innovision), as previously reported in detail (10, 14). In brief, the patient breathes into a closed-circuit rebreathing system composed of a respiratory valve system connected to a bag prefilled with oxygen (O_2_), ambient air and known small quantities of two different inert gases: the nitrous oxide (N_2_O) that is rapidly and completely absorbed by blood, and sulphur hexafluoride (SF_6_), which rests in the lung. Expiratory gas concentrations are continuously measured using a photoacoustic analyser. N_2_O concentration decreases during the rebreathing manoeuvre, with a rate proportional to PBF. SF_6_ allows the measurement of the volume of the lungs with a uniform distribution of N_2_O.

Effective dead space was calculated at each step by arranging the formula of VE = (863 × VCO_2_)/[PaCO_2_ × (1-VD/VT)] obtaining the VD/VT ratio, using VE, tidal volume (VT), and VCO_2_ measured in correspondence with arterial blood samples. PaCO_2_ was the arterial partial pressure of CO_2_ in blood samples, and 863 was a known constant. VE_DS_ was then calculated as (VD/VT) × (VT × Respiratory Rate), and alveolar ventilation (VA) was calculated as VE-VE_DS_ (4).

### Statistical analysis

Continuous variables are presented as mean ± standard deviation. Categorical variables are presented as frequencies and percentages. Analyses have been performed using IBM SPSS Statistics 27.

## Results

Eighteen patients from a cohort of PH patients regularly followed at our dedicated outpatient clinic were enrolled in the present study. Patients’ clinical characteristics and haemodynamic parameters are reported in Tables 1, 2, respectively.

**Table 1:** Demographics of the patients in the study.

Males/females	8/10
Age (years)	57 ± 17
BMI (kg/m^2^)	25 ± 6
NYHA class II (%)	13 (71%)
NYHA class III (%)	5 (29%)
PH group 1 (%)	12 (72%)
PH group 4 (%)	5 (28%)

BMI, body mass index; NYHA, New York Heart Association; PH, pulmonary hypertension.

**Table 2:** Haemodynamic parameters at right heart catheterization.

PAPs (mmHg)	57 ± 24
PAPd (mmHg)	21 ± 11
PAPm (mmHg)	36 ± 14
RAPm (mmHg)	4 ± 2
PVR (WU)	6.8 ± 5.0
PCWP (mmHg)	11 ± 4
CO (L/min)	4.4 ± 1.5
CI (L/min/m^2^)	2.5 ± 0.9

PAPs, systolic pulmonary arterial pressure; PAPd, diastolic pulmonary arterial pressure; PAPm, mean pulmonary arterial pressure; RAPm, mean right atrial pressure; PVR, pulmonary vascular resistance; PCWP, pulmonary capillary wedge pressure; CI, cardiac index; CO, cardiac output (by thermodilution).

Regarding the clinical classification of PH, 13 patients were in group 1 (9 idiopathic, 2 cases of PH secondary to scleroderma, and 2 cases secondary to corrected atrial septal defect). The remaining 5 patients belonged to group 4 (chronic thromboembolic pulmonary hypertension). Thirteen patients were in NYHA functional class II and the remaining 5 were in NYHA class III. At the time of the study, 9 patients were treated with a monotherapy (5 with an endothelin receptor antagonist [ERA], 2 with a phosphodiesterase type 5 inhibitor [PDE5 inhibitor] and 2 with a soluble guanylate cyclase stimulator [sGC stimulator]). Seven patients were receiving double therapy (6 with an ERA and a PDE5 inhibitor and 1 with an ERA and sGC stimulator). The remaining 2 patients were treated with a triple therapy composed of an ERA, a PDE5 inhibitor, and treprostinil.

Spirometry and CPET data obtained at day 1 (maximal CPET) are shown in Table 3. Aerobic capacity reduction, as demonstrated by the low predicted VO_2_ value, was observed. Moreover, mean PetCO_2_ values at rest and peak were low, while the mean VE/VCO_2_ slope was increased. No indirect signs of exercise-induced intracardiac shunt were observed for any case (15).

**Table 3:** Main standard spirometry and maximal CPET data.

FVC (L)	3.2 ± 1.0
FEV_1_ (L)	2.4 ± 0.9
FVC (% of predicted)	92 ± 16
FEV_1_ (% of predicted)	86 ± 15
Peak VO_2_ (ml/min)	1,163 ± 480
VO_2_ (% of Predicted)	69 ± 13
PetCO_2_ (mmHg) at rest	27 ± 6
PetCO_2_ (mmHg) at the peak	27 ± 7
VE/VCO_2_ slope	41 ± 16
RER	1.1 ± 0.1
Breathing reserve (%)	33 ± 13
SO_2_ basal (%)	97 ± 1
SO_2_ peak (%)	93 ± 3

Peak VO_2_, oxygen uptake at peak exercise; %PredVO_2_, percentage of predicted oxygen uptake; PetCO_2_, end-tidal carbon dioxide pressure; VE/VCO_2_, ventilatory equivalent of carbon dioxide; RER, respiratory exchange ratio; SO_2_, haemoglobin O_2_ saturation.

Mean values of CO obtained at day 2 CPET with PhysioFlow, PBF obtained by Innocor, and the intrapulmonary shunt (IP shunt) calculated as the difference between CO and PBF at rest and each exercise step are reported in Table 4. CO increased from 4.86 ± 1.24 L/min (rest) to 9.41 ± 2.63 L/min in the 3rd step. PBF rose from 3.81 ± 1.41 L/min to 7.21 ± 2.93 L/min. Consequently, CO to unventilated alveoli, which means intrapulmonary shunt, doubled from 1.05 ± 0.96 L/min to 2.21 ± 2.28 L/min. Therefore, at 60% of maximum workload, the intrapulmonary shunt represented approximately 20% of CO. We observed a relevant exercise-induced desaturation (from 95% to 89%) in 1 patient (group 1). In this patient, intrapulmonary shunt was approximatively double that observed in the other PH patients. Using the same protocol in normal subjects, percentage differences between CO measured by PhysioFlow and PBF were approximately 5%, 3%, 4%, and 2% at rest, and 20%, 40%, and 60% at peak exercise, respectively (data on file).

**Table 4:** Mean values of CO and shunt obtained with the different methods.

	Rest	1st step	2nd step	3rd step
CO PhysioFlow (L/min)	4.86 ± 1.24	6.37 ± 1.62	7.71 ± 2.02	9.41 ± 2.63
PBF (L/min)	3.81 ± 1.41	5.35 ± 2.14	6.03 ± 2.37	7.21 ± 2.93
IP shunt (L/min)	1.05 ± 0.96	1.02 ± 1.65	1.68 ± 2.13	2.21 ± 2.28
Workload (watts)	0	16 ± 7	33 ± 15	49 ± 22

CO, cardiac output; PBF, pulmonary blood flow; IP shunt, intrapulmonary shunt.

Steps 1, 2, and 3 refer to 20%, 40%, and 60% of the maximum workload of the patients’ CPET.

CPET parameters during the measurement of CO at the different steps are reported in Table 5. Throughout exercise, a constantly high VD/VT ratio was observed. On average, VE_DS_ increased during exercise, representing almost 38% of VE at the third step. Of note, the mean value of Δ(PaCO_2_-PetCO_2_) was elevated throughout exercise. The percentages of shunt flow over CO and VE_DS_ over VE at rest and during each exercise step are reported in Table 6.

**Table 5:** Descriptive analysis of respiratory and arterial blood gas data at each step of exercise.

	Rest	1st step	2nd step	3rd step
VE (L/min)	15.2 ± 5.2	23.5 ± 8.8	31.1 ± 8.7	41.1 ± 12.0
VA (L/min)	9.0 ± 2.0	15.3 ± 5.3	18.8 ± 7.3	25.4 ± 7.6
VD (L)	0.3 ± 0.2	0.4 ± 0.2	0.5 ± 0.2	0.5 ± 0.2
VE_DS_ (L/min)	6.0 ± 4.1	8.2 ± 5.7	12.3 ± 5.7	15.7 ± 7.4
VT (L)	0.8 ± 0.3	1.1 ± 0.4	1.3 ± 0.4	1.6 ± 0.5
VD/VT	0.4 ± 0.1	0.3 ± 0.2	0.4 ± 0.2	0.4 ± 0.1
VCO_2_ (L/min)	0.3 ± 0.1	0.6 ± 0.2	0.7 ± 0.3	1.0 ± 0.4
Δ(PaCO_2_-PetCO_2_)	3.8 ± 4.7	3.6 ± 4.7	5 ± 3.9	4.4 ± 4.0
PetCO_2_ (mmHg)	28.1 ± 5.8	28.7 ± 5.5	29.1 ± 6.1	28.5 ± 5.6
PaCO_2_ (mmHg)	31.9 ± 4.7	32.2 ± 5.2	33.6 ± 5.6	32.9 ± 4.0
RR (breath/min)	20 ± 6	22 ± 6	25 ± 6	29 ± 8
SpO_2_ BGA (%)	97 ± 2	97 ± 2	96 ± 3	96 ± 3

VE, pulmonary ventilation; VA, alveolar ventilation; VD, dead space volume; VE_DS_, dead space ventilation; VT, tidal volume; VD/VT, ratio of physiologic dead space to tidal volume; VCO_2_, carbon dioxide output; PetCO_2_, end-tidal carbon dioxide pressure; PaCO_2_, partial pressure of carbon dioxide; BGA, blood gas analysis.

**Table 6:** Percentages during the exercise steps: shunt over CO and VE_DS_ over VE.

	Rest	1st step	2nd step	3rd step
Shunt over CO (%)	22 ± 18	16 ± 22	21 ± 25	23 ± 24
VE_DS_ over VE (%)	38 ± 13	33 ± 16	40 ± 18	38 ± 13

CO, cardiac output; VE_DS_, dead space ventilation; VE, pulmonary ventilation.

## Discussion

The present study confirms our previous data regarding the behaviour of VE_DS_ in patients with PH^4^; indeed, in the set of PH patients in this study, VE_DS_ at rest and during exercise is approximately 40% of total VE. Furthermore, in this study, we observed that, during exercise, CO increase is blunted, with 80% of blood flow reaching the ventilated lung zone and 20% reaching the unventilated lung zone, a distribution that remains constant up to 60% of the peak workload. The present study is the first in which, on top of partitioning of ventilation, lung blood perfusion has been reported considering its two components, i.e., blood flow to ventilated and unventilated lung zones.

The PH population we studied consisted of 18 subjects with moderate group 1 and 4 PH and who could perform a symptom-limited maximal CPET. All were in stable clinical conditions, as shown by the haemodynamic, treatment strategy, and CPET data, with, on average, a modest arterial desaturation during exercise. Of note, patients with hypoxia at rest or documented extra pulmonary shunt were excluded.

The gold standard method for the evaluation of CO during exercise is the Fick method, albeit thermodilution is the most frequently used. Both are invasive methods that require the insertion of a catheter into the pulmonary artery (16). In recent years, two non-invasive methods of CO measurement have been validated in normal subjects and patients with different heart and pulmonary diseases, including PH: the inert gas rebreathing technique (Innocor) and the thoracic impedance system (PhysioFlow) (17–19). In particular, Innocor calculates the CO as the sum between PBF, which is blood flow participating in gas exchange at the alveolar level, and the intrapulmonary shunt. The latter is estimated using a formula that includes the estimation of alveolar oxygen tension and oxygen saturation in the systemic arteries. In a previous study, we observed that Innocor shunt estimation was not reliable in patients with peripheral arterial saturation values of <90%, a condition frequently reached during exercise in PH patients (17). Conversely, the reliability of the estimation of intrapulmonary shunt and therefore total CO during exercise in PH patients can be questioned. Moreover, at peak exercise, the rebreathing technique can be performed, albeit with some uncertainty and difficulty. Accordingly, in our protocol, four steady state conditions were evaluated: rest and three loaded conditions equal to 20%, 40%, and 60% of peak workload. Measurements were taken for PhysioFlow between 120 and 150 s and for Innocor between 150 and 180 s at each step when steady state conditions are likely reached. Indeed, CO measurements with the two techniques, which cannot be taken simultaneously due to them interfering with each other, were recorded during the last 60 s of each step in comparable loading conditions.

Instead, PhysioFlow is not influenced by peripheral saturation and measures changes in transthoracic impedance, calculating the CO using the product of heart rate, stroke volume index, and body surface area. Accordingly, total CO can be measured through impedance and flow to the ventilated lung zone using the inert gas technique. Therefore, CO to unventilated lung zones is the difference between the two measurements.

Both the inert gas rebreathing technique (Innocor) and the thoracic impedance system (PhysioFlow) have been extensively used and validated at rest and during exercise. With regard to the thoracic impedance CO measurements, old reports showed some inaccuracy in CO determination during exercise in heart failure patients (20). However, other studies have reported its applicability during exercise in various populations (21–24), and a recent study by Louvaris et al. (25) reported good accuracy for CO determination at rest and during exercise, including the peak, using thoracic impedance in COPD patients. With regard to pulmonary hypertension patients, a few studies have reported a reliable comparison of thoracic impedance vs. thermodilution at rest (19, 26); this datum was not confirmed in a further study (27). Data on the reliability of PBF measurements using the inert gas rebreathing technique are abundant (28–30), starting with the pioneering work by Lang et al. (31, 32). In our laboratory, we confirmed the reliability of the inert gas rebreathing technique for CO measurements vs. the Fick method and thermodilution at rest and during exercise in heart failure patients (10). Moreover, a few reports confirmed the reliability of the inert gas rebreathing technique for PBF determination in patients with lung diseases (33) and pulmonary hypertension (17).

The present combined methodology to measure CO and PBF is a new promising technique for assessing CO in PH patients, distinguishing between blood perfusion of the ventilated and unventilated lung zones. This double CO methodology may allow, in larger population studies, a better understanding of how the different pulmonary vasodilating drugs, which affect different biochemical pathways, work, or how, in this regard, the combination of different treatments works. Indeed, although some increase in blood flow to the unventilated lung zone, i.e., some treatment-induced hypoxia, may be a reasonable cost to pay in all PH treatment strategies, its amount remains relevant but still unknown information. It must be underlined that to explain the double CO methodology we present in this study, we modelled a simplification of the underlining physiology regarding blood flow to ventilated and unventilated lung zones. It is acknowledged that, in reality in PH, several lung zones are hypoventilated, which nevertheless, from a functional point of view, contributes to intrapulmonary shunt.

The present study has several limitations that should be acknowledged. First, we applied a submaximal exercise up to 60% of the peak workload. This was needed to have a prolonged steady state condition, at least in the final minute of exercise, to apply the double CO methodology. However, this excludes a relevant part of exercise from the analysis, including the peak, when a greater intrapulmonary shunt is likely, as shown by the progressive arterial oxygen desaturation frequently observed in PH patients during exercise. Second, we excluded by necessity patients with intracardiac shunt, the presence of which alters the physiological basis of the double CO methodology. Third, only cases with mild PH were included; therefore, we do not know how blood flow distribution in the lung behaves during exercise in patients with more severe PH. Similarly, we do not know whether, as it is likely to happen, the duration of pulmonary hypertension influences intrapulmonary blood flow distribution. Fourth, in this first study, the population we studied was small; therefore, the effect of different PH treatment strategies, albeit desirable, could not be assessed. Similarly, for the very same reason, we were not able to analyse separately group 1 and 4 PH patients or patients with and without drug treatment; albeit, a tendency to a greater shunt, both at rest and during exercise, was observed in group 1 patients. This datum however needs to be confirmed in a larger population. Fifth, assessment of the behaviour of the intrapulmonary distribution of blood flow during exercise using the double CO technique we described has never been applied in patients with different diseases. The present study describes the first application of this technique. Finally, both non-invasive techniques may be imprecise, and data on their repeatability are limited in the literature and this was not examined in the present study.

In conclusion, we showed for the first time that partitioning between CO to ventilated and unventilated lung zones is feasible during exercise, and blood flow to the unventilated lung zone is approximately 20% of total CO while VE_DS_ is approximately 40%. The data from this preliminary study suggests the present technique is a useful tool for assessing the effects of pulmonary vasodilating drugs on intrapulmonary shunt. However, the present data should be confirmed in a greater population of PH patients, including a larger number of patients for each PH group. Indeed, it could be interesting, in future studies, to assess the changes of pulmonary blood flow partitioning during follow up in relation to therapeutic changes and evaluate the impact of intrapulmonary shunt on the prognosis of PH patients.

## Data Availability

The raw data supporting the conclusions of this article will be made available by the authors, without undue reservation.
